# Modern Medical Ethics; or State Maxims in Medicine

**Published:** 1830-01-01

**Authors:** 


### Periscope;

OR,

### CIRCUMSPECTIVE REVIEW.

"Ore trahit quodcunque potest, atque addit acervo."

I.

### Modern Medical Ethics; or State Maxims in Medicine. By Philo-Ethicus, *Artium Magister*, &c.

(Chapter the First.)

Medical Ethics (in the modern sense) must be considered the most important branch of our professional studies, because it involves the *science* of life (a knowledge of human nature) and the *art* of turning that knowledge to the greatest possible advantage. Now it is very remarkable that, although this noble science (of life,) this useful *art*, has been cultivated with great success during the last twenty years, and is now brought to the highest degree of perfection, not a line has been written on the subject, or any code of instructions put on record for the benefit of the *rising* or the *falling* generation! It appears to be what lawyers call the lex non scripta:—it is perfectly well understood by adepts; but hitherto it has been thought incapable of taking a tangible shape, even under the creative power of the press. We believe this is partly true; for much of the noble art is, like the *tact* or even the skill of the physician, incommunicable by words. But still we hope to shew that there are fundamental maxims in medical ethics which will prove useful text-books for those who are desirous of making progress in the art. Some one has said, "ars tota in observationibus"—that is, medical practice consists entirely in the treatment of single cases. So in medical ethics, the whole consists of insulated maxims founded on observation. These maxims require no particular arrangement—at least we shall give them none—but we will set them down as they occur to our recollection, and solicit the assistance of our friends in augmenting the list from time to time. We shall commence with the medical man's initiation into *practice*. With the previous education, classical or professional, we have nothing to do. There are different opinions respecting the necessity for either; and we shall not attempt to unloose the Gordian knot.

### CHAP. I.

#### Maxim I.

Set up your carriage. Without this symbol you cannot *get into* practice—without it, you could not *get through* practice—and without it you should not *go out* of practice. To *get into* practice, let your carriage be elegant, your liveries splendid, your horses very fast goers. If they run over half a dozen hapless pedestrians annually, and your coachman is punished by the magistrates, all the better. Even an occasional *deodand* will be a *God-send* in the way of reputation. To *get through* practice, you may slacken your pace—reduce the breadth of the embroidery on your liveries—paint your carriage but once in three years—and exchange your blood horses for common jobs. To *get out* of practice, it is not essentially necessary to put down your carriage. There are many other auxiliaries which it would be useless to mention. The process is somewhat analogous to that of parturition—it is wonderful what Nature and Time will do in this way!

You should never be seen lolling about in your carriage "spying farlies" out of the windows. Always appear to be making notes of your appointments; and ever and anon call out to the coachman to quicken his pace. Be sure to have an ink-stand pinned in the front of the carriage, and keep the seat strewed with letters from your patients.

MAX. II.

DRESS and ADDRESS. Great attention should be paid to these, while *getting into* practice. When your reputation is firmly established, your dress may be slovenly even to malpropreté—and your *address* may be uncourteous even to rudeness, with considerable advantage. Strange as it may appear, it is yet a certain fact, that it is nearly as difficult to throw off as to acquire a well-earned fame in medicine. On the other hand, fame without a solid foundation is like a ship without ballast—liable to be upset by the first squall.

MAX. III.

Search the journals for a long catalogue of desperate cases, which you are to get carefully by heart, making them all terminate favourably under your own superintendance. With a minute retail and detail of these you are to entertain each of your patients during three-fourths of the time which you devote to the daily visit. You are also to narrate the same histories to every individual with whom you come in contact, professional or non-professional — so long as they have patience to listen to, or credulity to believe them. This maxim ranks next in respectability, and perhaps success, to the ingenious *patent one* of Dr. Eady.

MAX. IV.

Never appear at the Opera, theatre, public assembly, private party, or medical society, without a well-arranged plan of being called out—in other words—of being "particularly wanted," to attend some patient in great danger. If any noted personage can be prevailed upon to lend his or her name, as the *appellant* on such occasions, the patronage will be invaluable. The moment the call is made, you are to bustle forth, otherwise the advantage of a personal recognition may be lost.

MAX. V.

HOSPITALS and INFIRMARIES. There is now some discrepancy of opinion respecting the policy of connecting yourself with a public institution. If you are *very clever* you will hardly want such an auxiliary:—if *very much the reverse*, you may stand a chance of some unpleasant exposures. In all cases it is very proper to present yourself as a candidate—taking care to procure a long list of testimonials for the printed circular and for the public advertisement.* If defeated, you have "made yourself favourably known to the public," and are entered on the list for every future contest. If successful, a wide theatre for your talents and ingenuity is opened. As a matter of course, you become a public lecturer—and a necessary consequence is, a niche in the TEMPLE of FAME, i.e. the head of the first column in every morning newspaper. In this extended metropolis, it is essential that every facility should be given to students, who wish to attend your lectures;—and therefore the *particulars* are to be learnt, not only at the school where you teach, but at all the medical booksellers' shops—and especially at your *own residence*, which is to be carefully pointed out in the newspapers. But besides these announcements, you are frequently to advertise your lectures on a new and specific plan. Not only are all the *diseases* on which you descant to be minutely enumerated in the advertisement; but the principal *symptoms* of these diseases are to figure in the columns of the Times, Herald, and other fashionable papers. These are your golden advertisements.†

---

* There is not the slightest necessity even to announce your intention to canvass for an appointment to an hospital or dispensary, when any vacancy occurs. You have only to say that you will, *on a future occasion*, present yourself as a candidate. This is an excellent and legitimate advertisement.

† It is not necessary, nor is it at all expected, that lectures should be actually delivered by those who keep them constantly advertised. That is quite a separate concern.

You are also to publish, through every possible channel, a monthly or quarterly numerical view of your public practice, according to a peculiar plan of registry of your own. The following specimen will afford you some idea of the plan.

Of 1000 patients treated at——— during the month of———nine hundred were completely cured—75 were greatly relieved—15 absented themselves—9 were discharged incurable — and 1 died.

In visiting out-patients of the hospital or dispensary, you should always leave your carriage or cabriolet at the door of the best house in the neighbourhood of the patient. This will well reward you for the little additional walk.

MAX. VI.

You must, by all means, make a collection of diseased structures, by begging all morbid parts that your friends may meet with. It is not of the slightest importance that you should be acquainted with the histories of the cases. These specimens of *diseases*, or "BOTTLE IMPS," you are to keep ranged in the room where you see your patients, or in a neighbouring apartment, and you should take care to shew them to all your patients, telling them that these were the only cases which you failed to cure, in your extensive practice, and that they are now bottled up for the benefit of the living, as they enable you to detect diseases with unerring certainty. This is a measure of the very first importance.

MAX. VII.

Write a book—or rather get some literary hack to write one for you, and dedicate it, at once, to the general reader. Medical men have neither time to read, nor money to buy, the tentative essays of their contemporaries :—address yourself therefore boldly to the whims, prejudices, fears, and foibles of the public. In your book, there is no occasion to investigate principles, but only to display the superiority of your own practice. Let your work therefore be studded with desperate cases, all terminating favourably, after the first men in the profession had failed. Give no other names or places of residence except the Duke of A———, the Marquess of B———, the Earl of C———, and so on ; and never descend lower than an M.P. Interlard the cases with extracts of letters from your patients, describing their complaints, and the great efficacy of your medicines. Dedicate your work to some fashionable physician or surgeon, from whom you will be sure to receive a complimentary letter that will be very serviceable on many occasions afterwards.

Supposing (which is not very likely to be the case) that you have made any useful discovery in medicine or surgery; —you are not to be such a simpleton as to reveal it openly to your professional brethren, who would instantly take advantage of it, without thanking you for your candour. No. You are to manage this point with great care and caution. A complete concealment of the remedy would subject you to the imputation of quackery ;—but you may throw such obscurity about the preparation, the dose, the administration, and so forth, of the medicine, while, at the same time, you dilate so amply on its miraculous efficacy, as to draw to yourself the whole practice of the remedy. If it be a piece of surgery, as straightening a crooked spine, widening a narrow channel, removing a troublesome excrescence, or, in short, performing any operation, then you are to shew that a peculiar manual dexterity, which cannot be described in words, has given you a facility and success quite worthy the attention of the public.

MAX. VIII.

If you are dubious of success, become a violent sectarian or politician. You will then be sure of employment among one party. Half a loaf is better than no bread.

MAX. IX.

THE GRAND SECRET.—The key-stone maxim on which all the great principles of medical ethics rest, has not yet been stated. It is to occupy a great portion of your nightly studies and daily avocations. You should not move a step without it. It is to the medical practitioner what the compass is to the mariner—what the pillar of light was to the wandering Israelites. It consists in the constant habit or practice of EXTOLLING YOURSELF AND DEPRECIATING YOUR NEIGHBOUR. This is, fortunately, not only the most useful maxim, but it is that which is most easily put in execution, and has the widest field for its application. No day in the week, nor hour in the day, can pass without presenting you with abundant opportunities for working this grand engine of advancement. It has this advantage too, that it may be practised, by way of amusement, at those periods when you have no other kind of practice on hand. All your friends can assist you in this way, without opening their purses—and a gossiping female, with a long and nimble tongue, may go far to make your fortune.

It is of great importance, however, that you should acquire adroitness and tact in the exercise of this leading maxim. Gross self-flattery might draw on you ridicule—and open defamation of your neighbour might draw on you the harpies of the law. Thus, suppose you are called in to a dangerous case, where another practitioner has been before you. You are not to say, in the presence of competent witnesses, that your predecessor had murdered, or poisoned, or ruined the patient. For doing so £500, with costs, were paid not a thousand years ago. If you have a particle of expression in your countenance, you may by looks, and gestures, and tones, and monosyllables, effectually harrow up the feelings of the parents or friends, and convince them that the life of the patient has been endangered or lost by the practice hitherto pursued.

"Sunt verba et voces quibus hunc (vexare) dolorem."*

The less danger there is in the case, the more decidedly must you make it appear that there is *now* scarcely a chance of recovery—but nevertheless you will make one effort to save life. A cure performed under such desperate circumstances, will greatly spread your own fame, while it fulfils the other part of the grand maxim, by depreciating your neighbour.

In all cases, without exception, where you are separately applied to as the secondary or ternary consultant, you are to express your regret that you had not seen the case before it had gone thus far. By this expression you do not entirely violate the truth, and even if it comes to the ears of the former consultants, you may defend the expression, as one used by the very first authorities in the profession.

In consultations, and especially in this metropolis, it is necessary to be a little cautious how you express or insinuate disapprobation of your brethren—and LIONS from the country often get themselves into difficulties in this way, when first settling in London. Still, although it may not be prudent to assume any superiority on your own part, or inferiority on the part of your colleagues, you are never to lose sight of the principle, but to manœuvre so that the patient or friends may *infer* that superiority which you dare not openly *claim*. This may be done in a thousand ways, by a man of ingenuity. Thus, suppose you are called in when an acute inflammation has been subdued, or all but subdued, by active measures, and

---

* The writer of this article is now attending the wife of a tradesman, who had been under the care of a respectable practitioner in Southwark, and who recommended his patient to go into the country for a few weeks, giving her, at the same time, some prescriptions for her use. She went to a village, 14 miles from town; but not getting better, she sent for a practitioner of the place, and shewed him the prescriptions of the other gentleman. He did not mince the matter, but exclaimed at once, that she might just as well have been swallowing arsenic all the time, as the medicines she had been taking! She believed it—came back to town soon afterwards—and discarded her former medical attendant without his knowing why or wherefore! Nothing is more common than this practice.

yet where pain, irritability, or other unpleasant feelings remain. You are strongly to insist on an anodyne, which could not have been safely prescribed before. The consequence will be a tranquil night—blessings on the new doctor and his prescription—and as a necessary corollary, a reflexion on the previous treatment.

Suppose, on the other hand, some acute or subacute inflammatory action arises in the course of a chronic and obscure complaint, and you are called in at this juncture. You are immediately to recommend moderate depletion, (the measure, in fact, which was about to be employed by the previous attendants,) the consequence of which will be, a temporary amelioration of symptoms, and a conviction, on the part of the patient and friends, that this depletive measure ought to have been long before employed. If the chronic disease, on which the acute or subacute supervened, be of a necessarily fatal nature, you are to give pretty strong hopes to the family of recovery—especially if the prior attendants had expressed their doubts on this point. The falsification of your hopes by the final event, is not of the slightest consequence. You will have injured your colleagues, mean time, in the opinion of the friends, (for the last opinion is always considered the best,) and you will have plenty of time to modify your prognosis afterwards; and, as the fatal catastrophe approaches, to fling the blame on your neighbours, by insinuating that, had more active measures been early employed, the event would have been different. This is a first-rate maxim, and is one of great power when artfully executed.

If an opinion has been given by your colleague or colleagues as to the nature or seat of the disease, you are always to give an opinion somewhat different—and take care that the parents or friends of the patient know it. If no dissection takes place, you are triumphant, because you can maintain positively that you were right, and that the others were wrong. If a *post-mortem* examination is permitted, you must still shew your skill and dexterity by making the pathology correspond with the diagnosis. Nothing is more easy than this, to a man of parts and *pretensions*. Suppose, for example, that a man dies after you had pronounced that the disease was inflammation of the brain. When the scull-cap is removed, you are to knead the brain with your fingers, in the same way that a baker kneads dough in a trough—under the pretence that you are feeling for abscesses. On prosecuting the dissection, you will find some portions of brain softened down by the above process. These you are to scrape off on your scalpel, and triumphantly shew them round as portions of *suppurated brain*. It is of no consequence that there should be no injection of vessels, or other marks of inflammation. These have all disappeared before death, leaving the purulent matter to prove the correctness of your diagnosis. In short, there is no part of the body in which a fertile imagination and a good modicum of effrontery may not easily make out traces of disease for the purpose in question. And having once found or formed these, you are to declare that it is quite unnecessary to seek for causes in any other places, when they are so evident in the place predicted before death. If a further dissection be insisted on, and more morbid anatomy turns up, you are to ridicule the idea of the latter having any thing to do with the disease. All other morbid appearances than those which suit your purpose are to be voted occurrences in the agonies of death.

